# Social determinants of health associated with increased prevalence of childhood malnutrition in Africa

**DOI:** 10.3389/fnut.2024.1456089

**Published:** 2024-11-01

**Authors:** Samuel Faraday Saidu, Ramona Ann Danielson

**Affiliations:** Department of Public Health, North Dakota State University, Fargo, ND, United States

**Keywords:** stunting, underweight, political stability, water sanitation and hygiene (WaSH), literacy, health access and quality index, social determinants of health, child nutrition

## Abstract

**Introduction:**

Childhood malnutrition remains a critical public health concern in Africa, with significant long-term consequences for children’s growth, development, and overall health.

**Methods:**

This study examined the association between the prevalence of underweight and stunting of children <5 and six social determinants of health. Data were taken from publicly available data sources. After data quality criteria were met, data were analyzed for 50 African countries using descriptive statistics and one-way ANOVA. A quartile analysis was performed on each predictor variable. Countries were also analyzed according to five subregions.

**Results:**

The average prevalence of children <5 who were underweight was 14.5% and who were stunted was 26.8%. There were significant differences by region in the prevalence of underweight but not stunting, and in average access to basic sanitation services, basic drinking-water services, literacy rate, and HAQ index. The quartile analysis indicated basic sanitation services, prevalence of open defecation, basic drinking-water services, literacy rate, HAQ Index, and Political Stability Score were statistically significantly associated (*p* < 0.05) with underweight, while all of these except open defecation were associated (*p* < 0.05) with stunting.

**Discussion:**

This study emphasizes the variations in underweight and stunting prevalence, but also demonstrates patterns among how the risk for these child malnutrition outcomes are distributed. The results offer a multifaceted understanding of factors influencing childhood malnutrition. By focusing on key underlying social determinants of health, substantial improvements in nutritional outcomes may be achieved, ultimately enhancing the health and well-being of children across the African continent.

## Introduction

Childhood malnutrition remains a pressing public health issue across Africa, with adverse impacts on children’s growth, development, and overall well-being (1, 2). Recent studies have shed light on the widespread prevalence of underweight and stunting among African children (3–5). Underweight and stunting are common forms of malnutrition in many African countries, with serious long-term effects for children’s health and development. They are frequently signs of chronic malnutrition. Furthermore, underweight and stunting are public health priorities in many African countries due to their high prevalence and negative effects on child mortality, cognitive development, and future productivity (6, 7). The United Nations International Children’s Emergency Fund (UNICEF) and the World Health Organization (WHO) report reveals concerning statistics, indicating that in 2022, the global prevalence of underweight was reported at 12.3%, while the prevalence of stunting stood at 22.3% (8–10).

Malnutrition is believed to be a contributing factor in over one-third of all child deaths in Sub-Saharan Africa (11). Poverty is the major contributor to this issue, creating a vicious cycle of poverty, disease, and malnutrition (11). Other key factors include lack of education, especially among women, adverse climatic conditions, sociocultural barriers, and lack of government investment (11). Food insecurity is a major driver, with over 204 million people in Sub-Saharan Africa suffering from hunger as of 2019 (12). Certain groups are particularly vulnerable, including women, victims of conflict, the ill, and children under age 5 (11–13).

Social determinants of health (SDH) are conditions in the lives of people that affect their health outcomes that are not specifically medical (14). SDH related to basic amenities and environment include basic sanitation, which is vital for preventing diarrheal diseases and enhancing overall health outcomes by ensuring access to essential hygiene facilities (15, 16). Studies have found that children from households lacking basic sanitation facilities are 13% more likely to be stunted, and those without access to handwashing facilities are 27% more likely to be stunted. Improving access to safe water, sanitation, and hygiene (WaSH) facilities has been shown to have a significant impact on reducing underweight and stunting in children under age 5 across low-and middle-income countries, including in Africa (17). Progress on improving access to basic drinking water services in Africa has been slow, with only a few countries on track to achieve universal coverage by 2030 (18). Access to basic drinking-water services is another environmental SDH linked to childhood malnutrition. Inadequate access to clean water increases the risk of waterborne diseases and infections, which impair nutrient absorption and overall health in children, contributing to malnutrition and stunted growth (19). Improving WaSH could prevent many of these health issues, significantly reducing childhood morbidity and mortality associated with malnutrition (19). Open defecation contributes to childhood malnutrition by promoting the spread of infections and diarrheal diseases, which affect nutritional absorption and overall health, aggravating child undernutrition and stunting (20).

SDH related to education, access to services, and social protection are also important. Adult literacy is positively associated with improved childhood nutrition, as higher levels of education are linked to better health knowledge and practices that support child health and development (21). Health access and quality are important for understanding the capacity of health systems to address various health issues, including childhood malnutrition (22). Political instability and violence interrupt key services such as healthcare, sanitation, and food supply chains, increasing malnutrition (23). Children in politically unstable places are more likely to face food insecurity, limited healthcare access, and increased exposure to infectious diseases, all of which lead to greater malnutrition rates. Additionally, instability frequently displaces families, compromising children’s nutrition and health (23).

## Methods

### Sample

**Figure 1:**
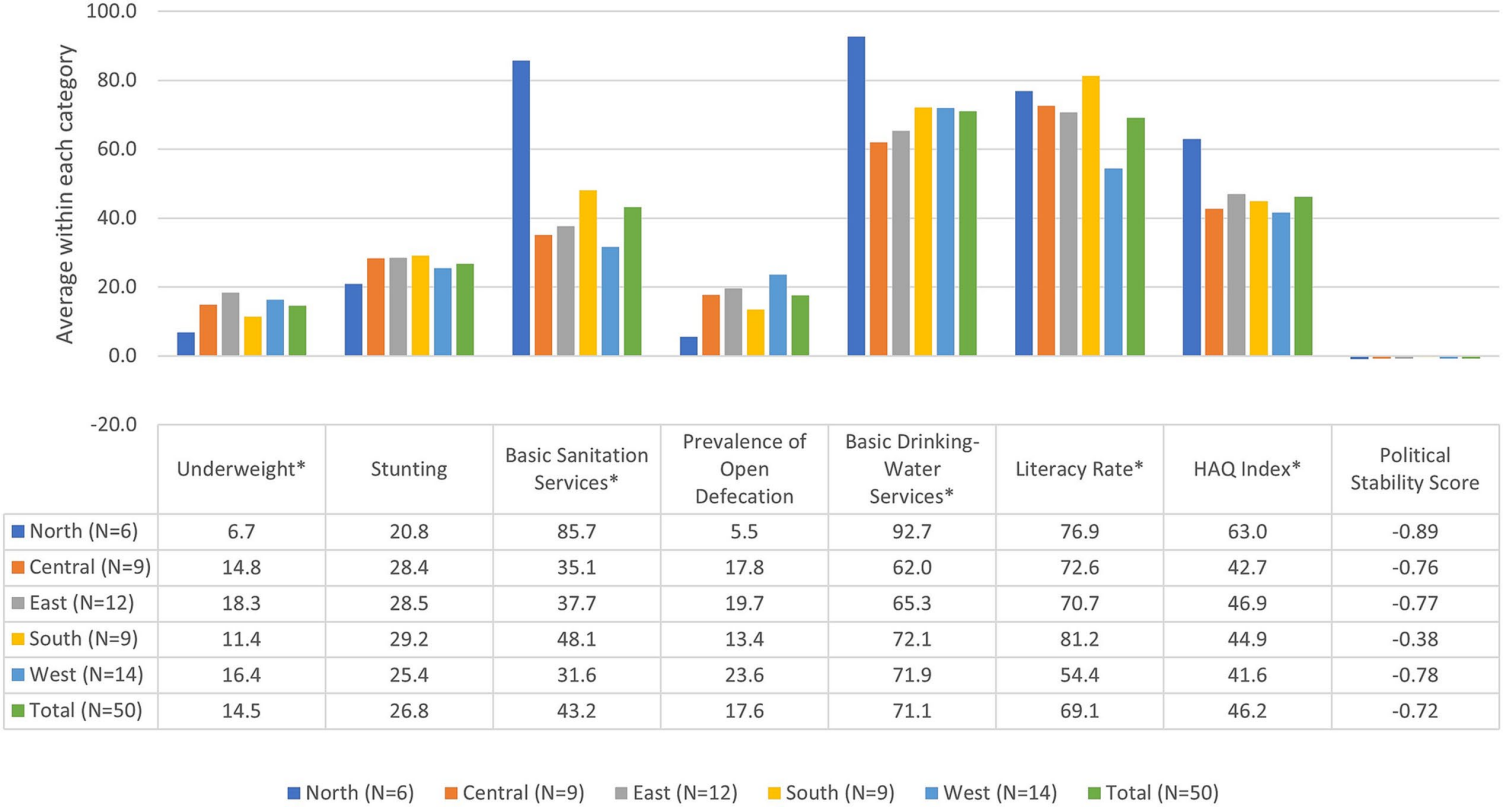
Scatterplot of prevalence of underweight for individual countries by region. *Significant at *p* < 0.05 using one-way ANOVA.

This study employed a quantitative approach, utilizing data from WHO Global Health Observatory, UNICEF Data, CIA World Factbook, the Global Change Data Lab’s Our World in Data, and World Bank Open Data. Countries with outdated information (older than 2010) were excluded (*N* = 4; Botswana, Cabo Verde, Mauritius, and Somalia) and no data were available in the data sources for the Sahrawi Arab Democratic Republic, resulting in a final sample size of 50 countries in Africa. Countries were categorized into regions according to the five subregions specified by the African Union (e.g., as shown in Figure 1; Supplementary Figure S1).

### Measures

#### Outcome variables

The prevalence of *underweight* children refers to the proportion of children under age 5 whose weight-for-age falls more than two standard deviations below the median for the international reference population aged 0 to 59 months (6). *Stunting*, a condition characterized by hindered progress and maturation in children, is often linked to factors such as insufficient nutrition, frequent illnesses, and inadequate mental and social stimulation. Children are considered stunted when their height falls more than two standard deviations below the median of the WHO Child Growth Standards for their age (7, 10).

#### Predictor variables

Six social determinants of health variables were examined as predictors in this study. *Basic sanitation services* (% of population with access to basic sanitation services) measures the population with access to the provision of facilities and services for the safe disposal of human urine and feces, such as flush toilets not shared with other households, toilets connected to piped sewer systems, pit latrines with slabs, or composting toilets (24). *Prevalence of open defecation* (% of population practicing open defecation) is the lack of toilet usage, excluding those using inadequate facilities like pit latrines without a slab (20). *Basic drinking-water services* (% of population using at least basic drinking-water services) measures the population accessing improved water sources within a 30-min round trip. It covers basic and safely managed drinking-water services, including piped water, boreholes, protected wells, springs, rainwater, and delivered water (17). *Literacy rate* (% of people age 15 and above) represents the proportion of individuals aged 15 and above who possess the ability to read and comprehend a brief, straightforward statement regarding their daily life (25). *Health access and quality index (HAQ Index)* is a measure that assesses the availability and quality of healthcare services across different countries and regions. The HAQ Index scores range from 0 to 100, with 0 representing the worst possible healthcare access and quality, and 100 representing the best (22). *Political stability and absence of violence/terrorism score (Political Stability Score)* assesses the perceived probability of political instability and/or violence, including terrorism, motivated by political factors. The scores range from-2.5 to 2.5, with higher positive values indicating greater political stability and absence of violence/terrorism (26).

### Analysis

SPSS version 29.0.2 was utilized for data analysis, employing descriptive statistics and one-way ANOVA. The 50 countries under study were segmented into four quartiles for each predictor; each quartile represented a different level of risk associated with that predictor variable. Quartile 1 (most risk) comprised the countries exhibiting the highest prevalence of each respective outcome variable when assessed against the respective predictor variable. These countries faced the most significant challenges and vulnerabilities concerning the outcome variable under consideration. Quartile 4 (least risk) comprised the countries with the lowest prevalence of each respective outcome variable when assessed against the respective predictor variable. These countries were considered the least vulnerable and faced more favorable circumstances concerning the outcome variables. Quartile analysis compared averages among the two outcome variables across the predictor variables.

## Results

**Figure 2:**
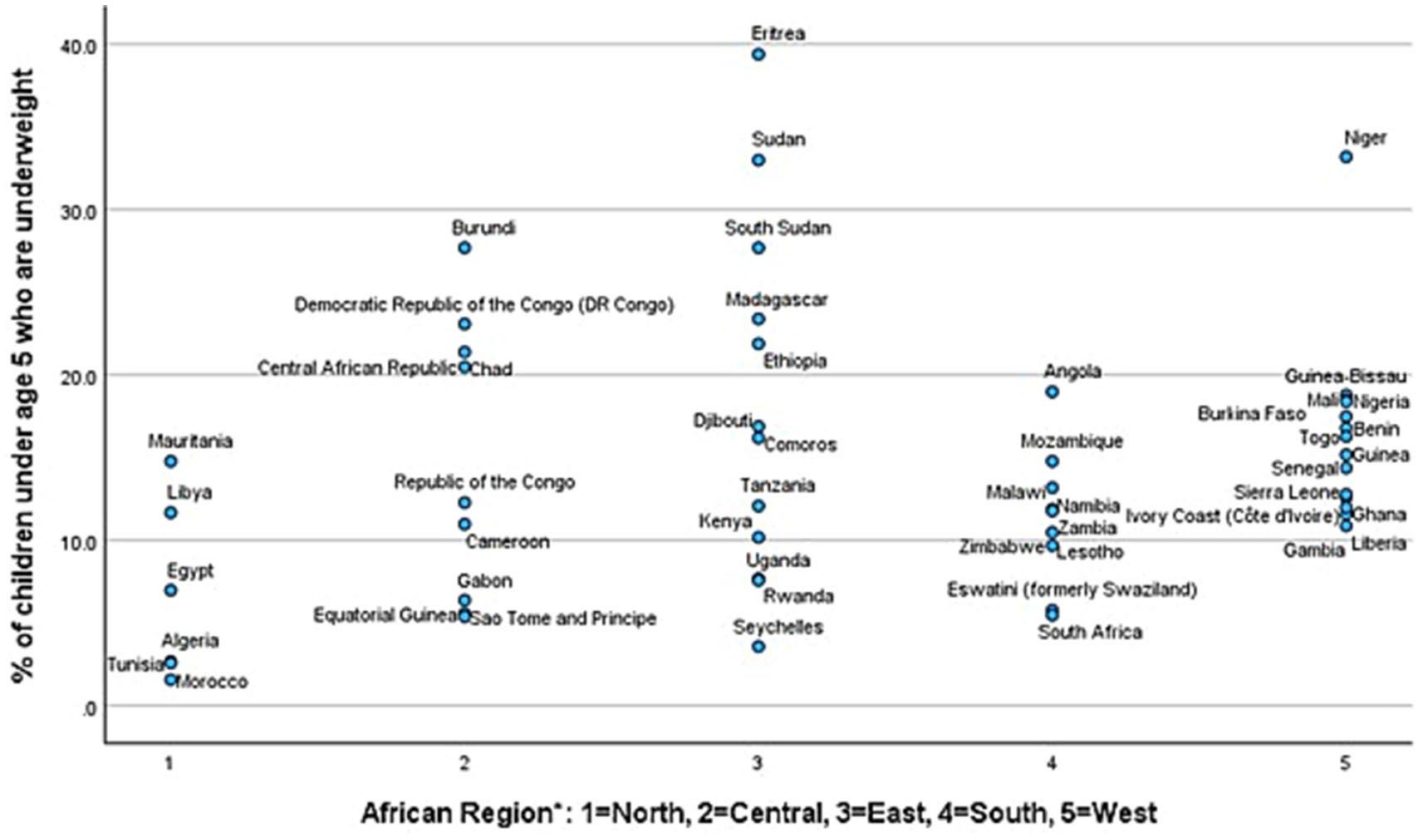
Means of outcome and predictor variables by region. *Significant at *p* < 0.05 using one-way ANOVA.

The frequency of underweight and stunting varied significantly throughout the 50 African countries studied (see Table 1; Figure 2). The average underweight percentage among the countries was 14.5%, ranging from 1.6 to 39.4%. Regionally, average percentages ranged from 6.7% for Northern countries to 18.3% for Eastern countries; differences in prevalence of underweight by region were statistically significant at *p* < 0.05. The average proportion of stunting was 26.8%, ranging from 7.3 to 56.4%. Figure 1 provides a scatterplot of prevalence of underweight in individual countries by region. Regionally, average percentages ranged from 20.8% for Northern countries to 29.2% for Southern countries; however, differences in prevalence of stunting by region were not statistically significant. Supplementary Figure S1 provides a scatterplot of prevalence of stunting in individual countries by region. These results demonstrate high variation in underweight and stunting prevalence across the continent.

**Table 1:** Descriptive statistics for outcome and predictor variables in analysis of childhood malnutrition in African Nations.

Variable	Mean	Range	
		Low	High
Outcome variables			
Underweight (% of children under age 5)	14.5%	1.6%	39.4%
Stunting (% of children under age 5)	26.8%	7.3%	56.4%
Predictor variables			
Basic sanitation services (% of population with access to basic sanitation services)	43.2%	9.0%	100.0%
Prevalence of open defecation (% of population practicing open defecation)	17.6%	1.0%	67.0%
Basic drinking-water services (% of population using at least basic drinking-water services)	71.1%	36.0%	100.0%
Literacy rate (% of adults age 15 and above)	69.1%	27.3%	96.2%
Health access and quality index (HAQ Index; index ranges from 0 to 100)	46.2	26.5	70.1
Political stability and absence of violence/terrorism score (Political Stability Score; score ranges from −2.5 to 2.5)	−0.72	−2.48	0.76

Among the predictor variables, access to basic services varied considerably (see Table 1; Figure 2). Averaging the proportions for each country, the average proportion of the population with access to basic sanitation services was 43.2%, ranging widely from almost no access at 9.0% to complete access at 100.0%. Differences by region were statistically significant at *p* < 0.05; the average percentages of access to basic sanitation services ranged from 85.7% for Northern countries to 31.6% in Western countries. The average proportion of the population practicing open defecation was 17.6%, ranging widely from almost no open defecation at 1.0% to two-thirds of the population practicing open defecation at 67.0%. Differences in prevalence of open defecation by region were not statistically significant. The average usage of basic drinking water services stood at 71.1%, with access proportions varying from just over one-third (36.0%) to complete access (100.0%). Differences by region were statistically significant at *p* < 0.05; the average percentages of usage of basic drinking water services ranged from 92.7% for Northern countries to 62.0% for Central countries. The average literacy rate was 69.1%, ranging from about one-fourth of the population 15 and older being literate (27.3%) to nearly complete literacy (96.2%). Differences by region were statistically significant at *p* < 0.05; the average percentages of population literacy ranged from 81.2% for Southern countries to 54.4% for Western countries. The average HAQ Index was 46.2, ranging from lower quality at 26.5 to higher quality at 70.1. Differences by region were statistically significant at p < 0.05; the average HAQ index ranged from 63.0 for Northern countries to 41.6 for Western countries. The Political Stability Score averaged-0.72 across the 50 African countries, and demonstrated substantial variation, with some countries assessed at being at great risk with a score of-2.50 and others assessed as experiencing relatively more stability at 0.76. Differences in political stability by region were not statistically significant.

Supplementary Figure S2 illustrates the variation in underweight prevalence across quartiles for each of the predictor variables. As the level of risk increased by quartile for basic sanitation services, prevalence of open defecation, basic drinking-water services, literacy rate, the HAQ Index, and Political Stability Score, the prevalence of underweight among children under age 5 saw a statistically significant increase (*p* < 0.05) (see Table 2). The spread in the prevalence of underweight between Quartile 1 (most risk) and Quartile 4 (least risk) was 14.9 percentage points for basic sanitation services, 13.7 for basic drinking-water services, and 13.5 for HAQ Index. The risk ratio of prevalence of underweight among children under age 5 was more than twice the risk for Quartile 1 compared to Quartile 4 for all of the SDH; the risk ratio was 3.1 times greater for basic sanitation services and 2.8 times higher for basic drinking-water services. The distributions of prevalence of underweight for each African country by quartile are shown in Supplementary Figure S3 for basic sanitation services, Supplementary Figure S4 for prevalence of open defecation, Supplementary Figure S5 for access to basic drinking-water services, Supplementary Figure S6 for literacy, Supplementary Figure S7 for HAQ Index, and Supplementary Figure S8 for political stability score.

**Table 2:** Difference in risk for underweight and stunting among children under age 5 in African Nations between highest and lowest risk quartiles for respective social determinants of health.

Social determinant of health	Underweight		Stunting	
	% Risk difference	Risk ratio	% Risk difference	Risk ratio
Basic sanitation services*	14.9	3.1	14.5	1.7
Prevalence of open defecation*	11.4	2.3	NA	NA
Basic drinking-water services*	13.7	2.8	18.8	2.1
Literacy rate*	12.0	2.6	13.2	1.7
HAQ Index*	13.5	2.4	17.4	1.8
Political Stability Score*	11.6	2.2	16.8	1.9

*Significant at *p* < 0.05 using one-way ANOVA.

Supplementary Figure S9 displays the variation in stunting prevalence across quartiles for each of the predictor variables. As the level of risk increased by quartile for basic sanitation services, basic drinking-water services, literacy rate, the HAQ Index, and Political Stability Score, the prevalence of stunting saw a statistically significant increase (*p* < 0.05) (see Table 2). However, prevalence of open defecation did not show a significant association with stunting. The spread in the prevalence of stunting between Quartile 1 (most risk) and Quartile 4 (least risk) was 18.8 percentage points for basic drinking-water services, 17.4 for HAQ Index, and 16.8 for Political Stability Score. The risk ratio of prevalence of stunting among children under age 5 was at least 1.7 times greater the risk for Quartile 1 compared to Quartile 4 for all of the SDH; the risk ratio was 2.1 times greater for basic drinking-water services. The distributions of prevalence of stunting for each African country by quartile are shown in Supplementary Figure S10 for basic sanitation services, Supplementary Figure S11 for prevalence of open defecation, Supplementary Figure S12 for access to basic drinking-water services, Supplementary Figure S13 for literacy, Supplementary Figure S14 for HAQ Index, and Supplementary Figure S15 for political stability score.

## Discussion

The findings of this study emphasize the significant variations in underweight and stunting prevalence among children under age 5 across 50 African countries, but also demonstrate patterns among how the risk for these child malnutrition outcomes are distributed. The analysis highlights that important SDH—basic sanitation services, prevalence of open defecation, basic drinking-water services, literacy, health access and quality, and political stability and absence of violence/terrorism—are significantly associated with the prevalence of underweight and stunting among children under age 5 in Africa. These results suggest that improving these determinants could potentially reduce rates of underweight and stunting among children in Africa.

The results of this study highlight the importance of improving key SDH to reduce the prevalence of childhood malnutrition in Africa. The significant associations observed between the SDH examined in this study and childhood underweight and stunting prevalence highlight the need to address underlying factors in addition to efforts that directly address food access. By focusing on these critical areas, substantial improvements in nutritional outcomes can be achieved, ultimately enhancing the health and well-being of children across the continent. In this spirit, recommendations for efforts to address childhood malnutrition include:

- *Ensuring community empowerment and participation*: Empowering local communities to participate in decision-making processes regarding health interventions can lead to more effective and sustainable outcomes. Community engagement initiatives, such as participatory planning and community health committees, can help ensure that interventions are culturally appropriate and address the specific needs of each community (27, 28).
- *Targeting interventions for vulnerable groups*: Special attention should be given to vulnerable populations, including women, children under age 5, victims of conflict, and marginalized communities. Tailored interventions that address the specific needs and challenges faced by these groups can help mitigate the impact of SDH on childhood malnutrition (29).
- *Improving access to basic sanitation services*: Concerted efforts should be directed toward initiatives aimed at improving access to basic sanitation facilities across the region. By prioritizing interventions that target households lacking adequate sanitation infrastructure, such as proper toilets and handwashing facilities, communities can significantly mitigate the risk of diarrheal diseases and improve overall health outcomes among children (30).
- *Eradicating open defecation*: Investing in infrastructure development and community education programs is essential to promote proper sanitation practices and reduce the spread of infectious diseases, ultimately safeguarding children’s health and well-being. By prioritizing initiatives to eradicate open defecation and ensuring access to adequate toilet facilities, stakeholders can create healthier environments for children to thrive and reduce the prevalence of childhood malnutrition across the region (31).
- *Expanding access to basic drinking-water services*: Government and other humanitarian organizations should focus efforts on expanding access to safe drinking water sources within a reasonable distance, particularly in rural and underserved areas, and supporting initiatives aimed at achieving universal coverage of basic drinking-water services by 2030 to improve child health outcomes and reduce malnutrition rates (32).
- *Investing in education*: Governments should prioritize education initiatives, particularly adult literacy programs, to empower communities with knowledge and skills that support child health and nutrition. Higher levels of literacy among adults are associated with better health practices and can contribute to breaking the cycle of poverty and malnutrition (33).
- *Investing in early childhood development programs*: Investing in early childhood development programs, including early childhood education, nutrition counseling, and parental support, can have long-term benefits for children’s health and development. These programs can help mitigate the impact of SDH on childhood malnutrition by providing children with a strong foundation for healthy growth and development (34).
- *Strengthening healthcare systems*: Efforts to enhance healthcare access and quality, as measured by the HAQ Index, are crucial for addressing childhood malnutrition. This includes improving infrastructure, increasing healthcare workforce capacity, and ensuring the availability and equitable accessibility of essential services such as maternal and child health care, immunizations, and nutrition counseling (35).
- *Promoting political stability*: Addressing political instability and violence is essential for creating an environment conducive to sustainable development and improving living standards. Governments, regional organizations, and the international community should work together to promote peace, security, and good governance in African countries, which are fundamental for achieving progress in health and development goals (36).
- *Investing in research and monitoring*: Continued research and monitoring are essential for understanding the evolving dynamics of childhood malnutrition and evaluating the effectiveness of interventions. Governments, academic institutions, and international organizations should prioritize research on SDH and their impact on childhood malnutrition, as well as invest in robust monitoring and evaluation systems to track progress and identify areas for improvement (37).

## Strengths and limitations

This study boasts several strengths, including the use of comprehensive data from reputable sources, ensuring robust and reliable analysis. The inclusion of 50 countries provides a broad overview of the situation across different African contexts, enhancing the generalizability of the findings. Furthermore, by examining various SDH, the study offers a multifaceted understanding of factors influencing childhood malnutrition. However, there are notable limitations. The exclusion of countries with missing or outdated data may limit the comprehensiveness of the analysis. Additionally, the cross-sectional design of the study does not allow for establishing causal relationships between SDH and the prevalence of the outcomes.

## Data Availability

Publicly available datasets were analyzed in this study. This data can be found at: https://www.who.int/data/gho, https://www.cia.gov/the-world-factbook/, https://ourworldindata.org/, https://data.unicef.org/topic/nutrition/malnutrition/, https://data.worldbank.org/.

## Supplementary material

SUPPLEMENTARY FIGURE S1 Scatterplot of prevalence of stunting for individual countries by region. Differences were not significant using one-way ANOVA.

SUPPLEMENTARY FIGURE S2 The relationship of social determinants of health with underweight among children under age 5 in African Nations. *Significant at *p* < 0.05 using one-way ANOVA.

SUPPLEMENTARY FIGURE S3 Scatterplot of prevalence of underweight for each African country by quartiles for basic sanitation services. *Significant at *p* < 0.05 using one-way ANOVA.

SUPPLEMENTARY FIGURE S4 Scatterplot of prevalence of underweight for each African country by quartiles for prevalence of open defecation. *Significant at *p* < 0.05 using one-way ANOVA.

SUPPLEMENTARY FIGURE S5 Scatterplot of prevalence of underweight for each African country by quartiles for access to basic drinking-water services. *Significant at *p* < 0.05 using one-way ANOVA.

SUPPLEMENTARY FIGURE S6 Scatterplot of prevalence of underweight for each African country by quartiles for literacy. *Significant at *p* < 0.05 using one-way ANOVA.

SUPPLEMENTARY FIGURE S7 Scatterplot of prevalence of underweight for each African country by quartiles for HAQ index. *Significant at *p* < 0.05 using one-way ANOVA.

SUPPLEMENTARY FIGURE S8 Scatterplot of prevalence of underweight for each African country by quartiles for political stability score. *Significant at *p* < 0.05 using one-way ANOVA.

SUPPLEMENTARY FIGURE S9 The relationship of social determinants of health with stunting among children under age 5 in African Nations. *Significant at *p* < 0.05 using one-way ANOVA.

SUPPLEMENTARY FIGURE S10 Scatterplot of prevalence of stunting for each African country by quartiles for basic sanitation services. *Significant at *p* < 0.05 using one-way ANOVA.

SUPPLEMENTARY FIGURE S11 Scatterplot of prevalence of stunting for each African country by quartiles for prevalence of open defecation. Differences were not significant using one-way ANOVA.

SUPPLEMENTARY FIGURE S12 Scatterplot of prevalence of stunting for each African country by quartiles for access to basic drinking-water services. *Significant at *p* < 0.05 using one-way ANOVA.

SUPPLEMENTARY FIGURE S13 Scatterplot of prevalence of stunting for each African country by quartiles for literacy. *Significant at *p* < 0.05 using one-way ANOVA.

SUPPLEMENTARY FIGURE S14 Scatterplot of prevalence of stunting for each African country by quartiles for HAQ index. *Significant at *p* < 0.05 using one-way ANOVA.

SUPPLEMENTARY FIGURE S15 Scatterplot of prevalence of stunting for each African country by quartiles for political stability score. *Significant at *p* < 0.05 using one-way ANOVA.
