# Supplementary figures and images for: Social determinants of health associated with increased prevalence of childhood malnutrition in Africa
Source: Front Nutr. 2024 Nov 1;11:1456089. doi: 10.3389/fnut.2024.1456089 (PMC11565952; doi:10.3389/fnut.2024.1456089)

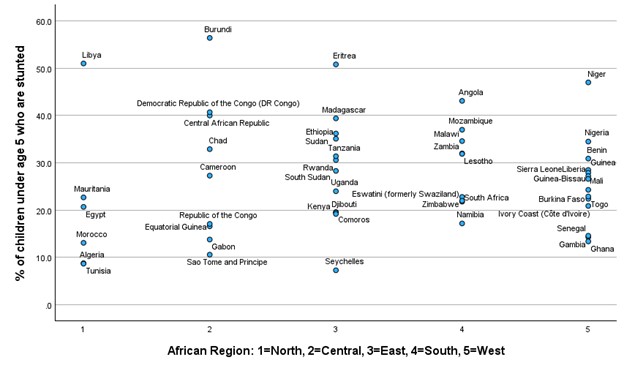

Supplement: SUPPLEMENTARY FIGURE S1 — Scatterplot of prevalence of stunting for individual countries by region. Differences were not significant using one-way ANOVA. [file Image_1.JPEG]

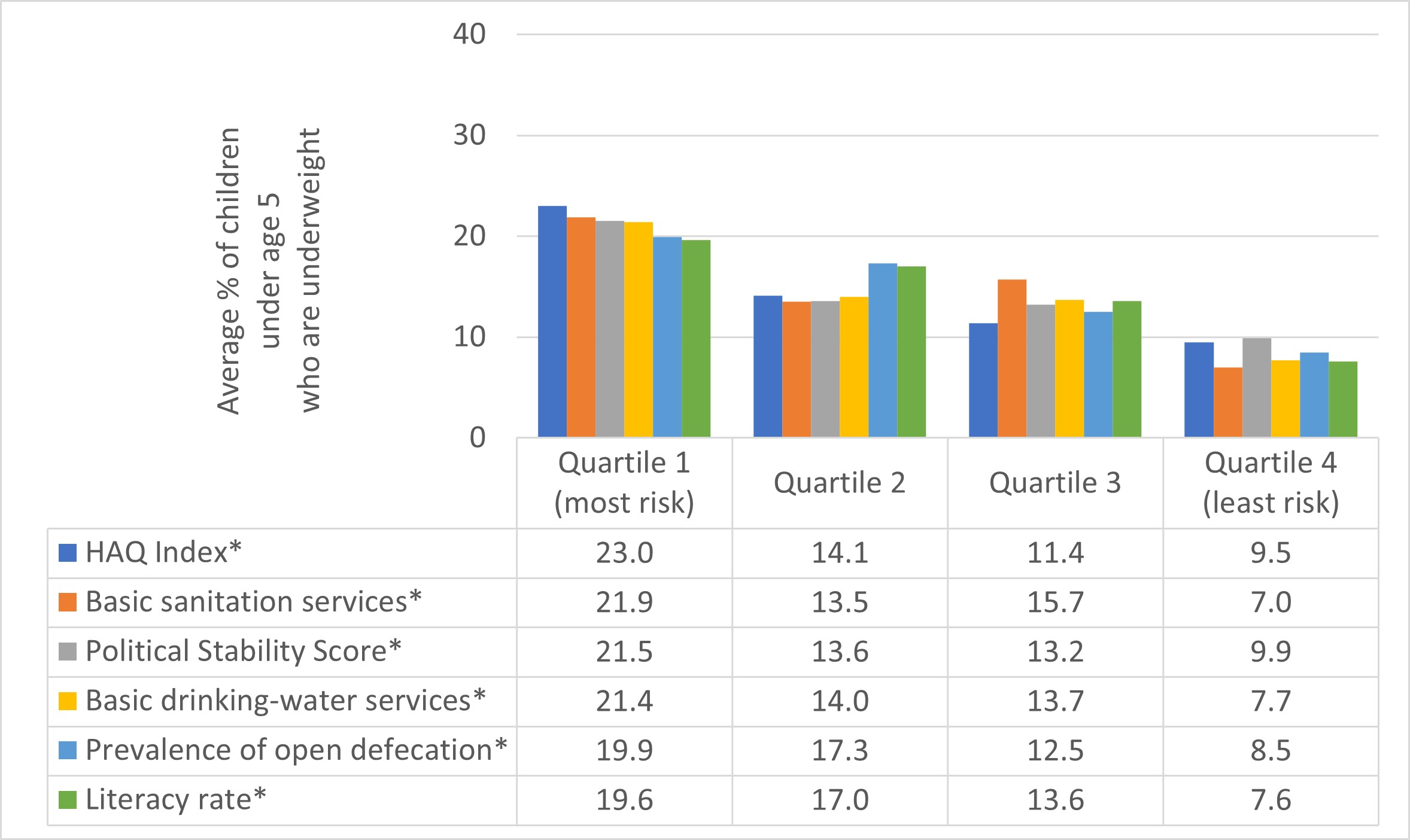

Supplement: SUPPLEMENTARY FIGURE S2 — The relationship of social determinants of health with underweight among children under age 5 in African Nations. *Significant at p < 0.05 using one-way ANOVA. [file Image_2.JPEG]

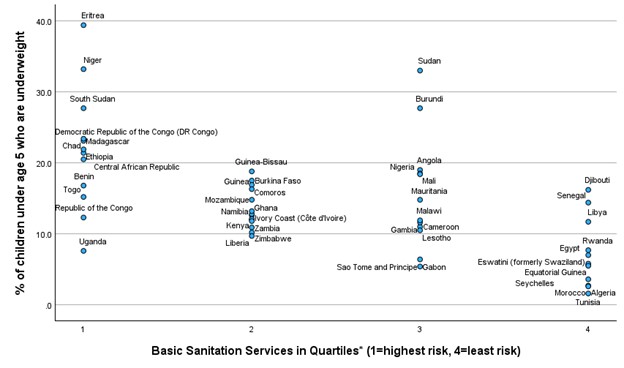

Supplement: SUPPLEMENTARY FIGURE S3 — Scatterplot of prevalence of underweight for each African country by quartiles for basic sanitation services. *Significant at p < 0.05 using one-way ANOVA. [file Image_3.JPEG]

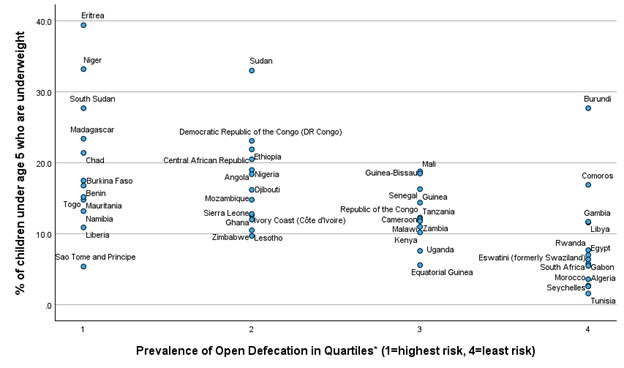

Supplement: SUPPLEMENTARY FIGURE S4 — Scatterplot of prevalence of underweight for each African country by quartiles for prevalence of open defecation. *Significant at p < 0.05 using one-way ANOVA. [file Image_4.JPEG]

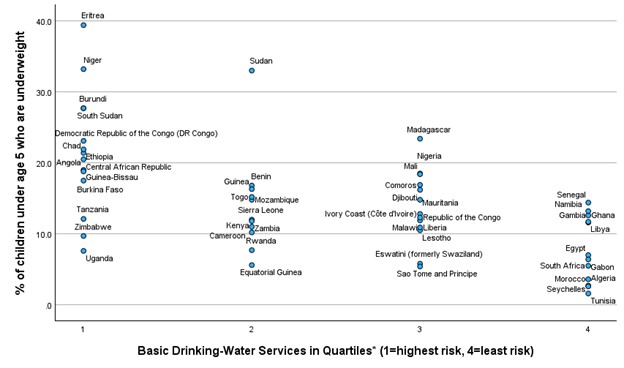

Supplement: SUPPLEMENTARY FIGURE S5 — Scatterplot of prevalence of underweight for each African country by quartiles for access to basic drinking-water services. *Significant at p < 0.05 using one-way ANOVA. [file Image_5.JPEG]

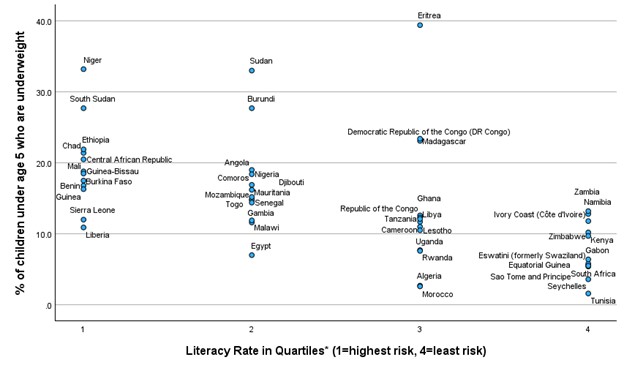

Supplement: SUPPLEMENTARY FIGURE S6 — Scatterplot of prevalence of underweight for each African country by quartiles for literacy. *Significant at p < 0.05 using one-way ANOVA. [file Image_6.JPEG]

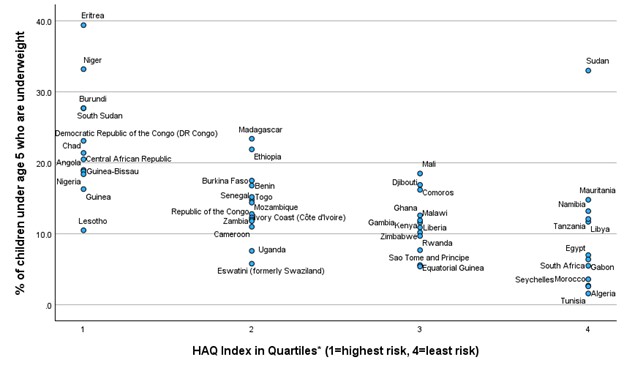

Supplement: SUPPLEMENTARY FIGURE S7 — Scatterplot of prevalence of underweight for each African country by quartiles for HAQ index. *Significant at p < 0.05 using one-way ANOVA. [file Image_7.JPEG]

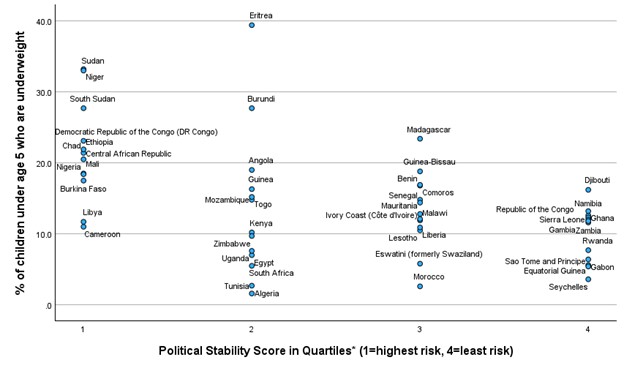

Supplement: SUPPLEMENTARY FIGURE S8 — Scatterplot of prevalence of underweight for each African country by quartiles for political stability score. *Significant at p < 0.05 using one-way ANOVA. [file Image_8.JPEG]

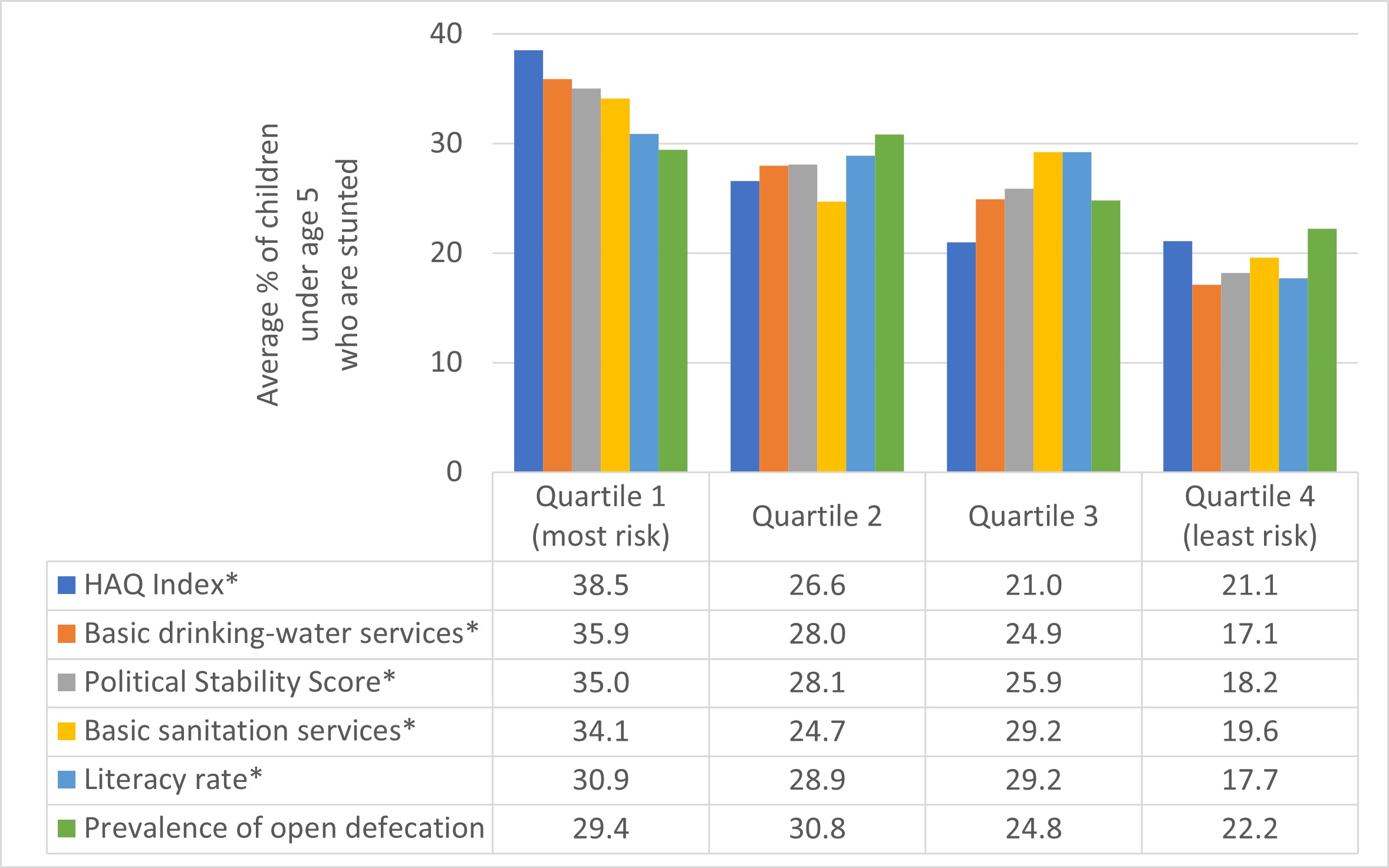

Supplement: SUPPLEMENTARY FIGURE S9 — The relationship of social determinants of health with stunting among children under age 5 in African Nations. *Significant at p < 0.05 using one-way ANOVA. [file Image_9.JPEG]

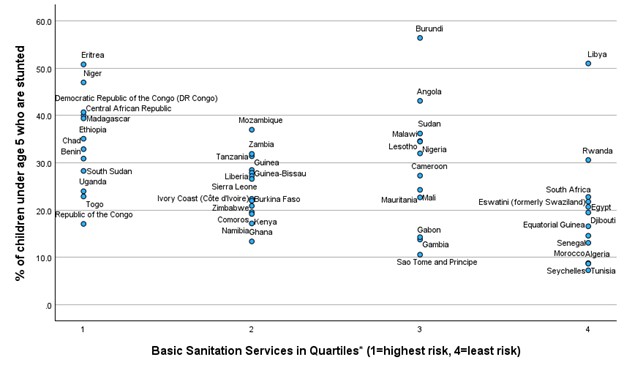

Supplement: SUPPLEMENTARY FIGURE S10 — Scatterplot of prevalence of stunting for each African country by quartiles for basic sanitation services. *Significant at p < 0.05 using one-way ANOVA. [file Image_10.JPEG]

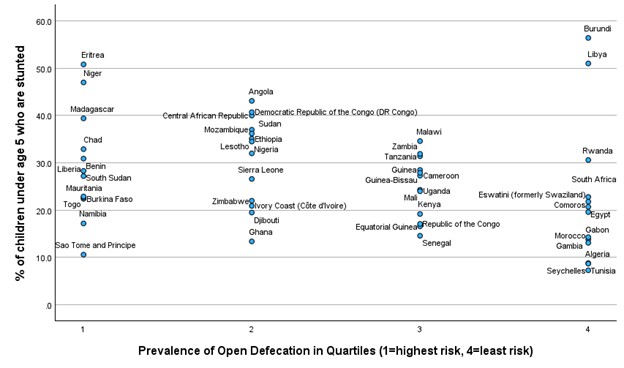

Supplement: SUPPLEMENTARY FIGURE S11 — Scatterplot of prevalence of stunting for each African country by quartiles for prevalence of open defecation. Differences were not significant using one-way ANOVA. [file Image_11.JPEG]

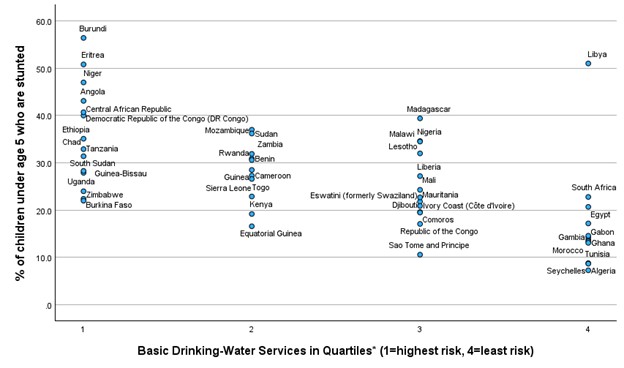

Supplement: SUPPLEMENTARY FIGURE S12 — Scatterplot of prevalence of stunting for each African country by quartiles for access to basic drinking-water services. *Significant at p < 0.05 using one-way ANOVA. [file Image_12.JPEG]

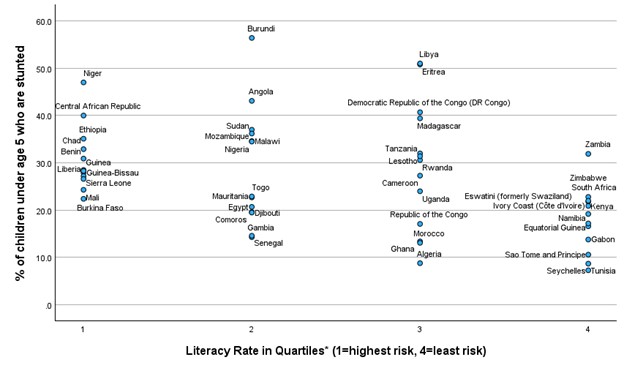

Supplement: SUPPLEMENTARY FIGURE S13 — Scatterplot of prevalence of stunting for each African country by quartiles for literacy. *Significant at p < 0.05 using one-way ANOVA. [file Image_13.JPEG]

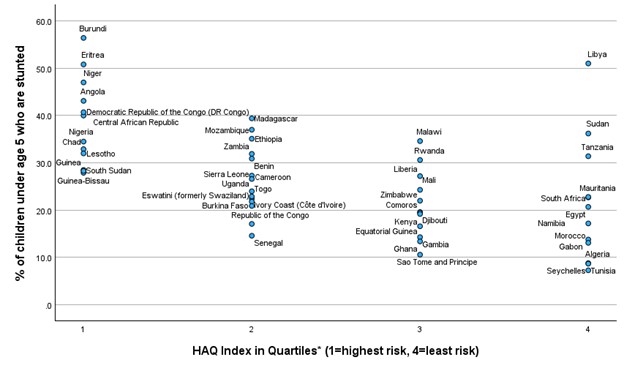

Supplement: SUPPLEMENTARY FIGURE S14 — Scatterplot of prevalence of stunting for each African country by quartiles for HAQ index. *Significant at p < 0.05 using one-way ANOVA. [file Image_14.JPEG]

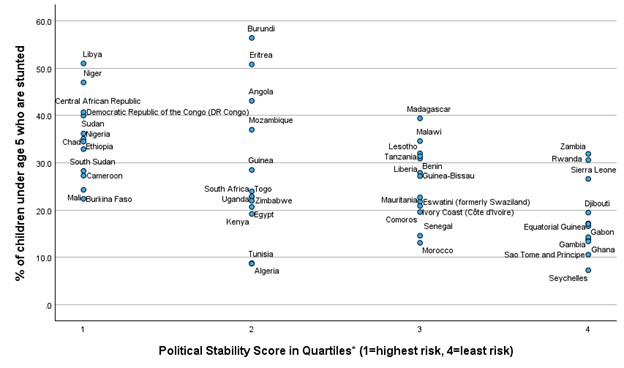

Supplement: SUPPLEMENTARY FIGURE S15 — Scatterplot of prevalence of stunting for each African country by quartiles for political stability score. *Significant at p < 0.05 using one-way ANOVA. [file Image_15.JPEG]
